# Acute Systemic Sclerosis-Associated Cardiomyopathy That Improved With Glucocorticoids and Cyclophosphamide

**DOI:** 10.1016/j.jaccas.2024.102948

**Published:** 2025-02-19

**Authors:** Andrew A. Gustafson, Katherine V. Trinh, Jon W. Lomasney, Sanjiv J. Shah, Monique E. Hinchcliff

**Affiliations:** a Northwestern University Feinberg School of Medicine, Division of Hospital Medicine, Northwestern University Feinberg School of Medicine, Chicago, Illinois, USA; b Department of Pathology, Northwestern University Feinberg School of Medicine, Chicago, Illinois, USA; c Department of Pharmacology, Northwestern University Feinberg School of Medicine, Chicago, Illinois, USA; d Department of Medicine, Division of Cardiology, Northwestern University Feinberg School of Medicine Northwestern University Feinberg School of Medicine, Chicago, Illinois, USA; e Department of Internal Medicine, Section of Rheumatology, Allergy & Immunology, Yale School of Medicine, New Haven, Connecticut, USA

**Keywords:** cardiac MRI, cyclophosphamide, myocarditis, myopericarditis, scleroderma, systemic sclerosis

## Abstract

**Background:**

Systemic sclerosis (SSc) cardiomyopathy has a prevalence of 7 to 39% and is associated with increased mortality. Despite this, little evidence informs SSc cardiomyopathy treatment.

**Case Summary:**

We present a patient with diffuse cutaneous SSc with acute heart failure. Extensive workup supported a diagnosis of SSc myopericarditis, although endomyocardial biopsies were unrevealing. She received intravenous cyclophosphamide and glucocorticoids and achieved significant and prolonged recovery.

**Discussion:**

Our patient presented with systolic dysfunction as opposed to diastolic dysfunction that is more typical in patients with SSc-cardiomyopathy. Endomyocardial biopsies lacked T-lymphocyte infiltration that may be due to sampling error because >17 samples are needed to diagnose myocarditis in >80% of cases.

## Graphical Abstract

**Figure:**
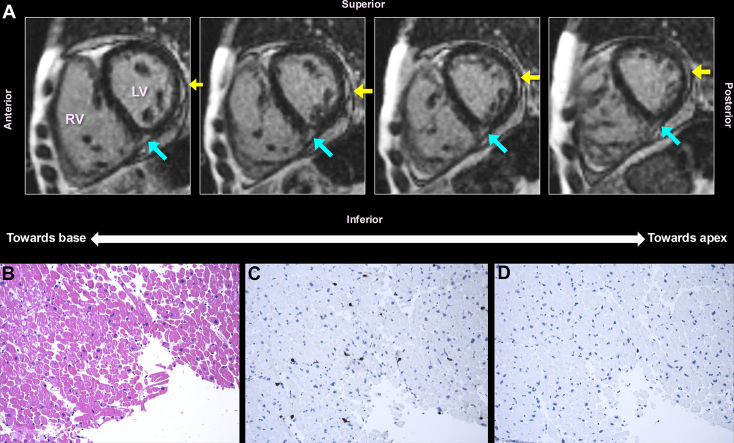

A 29-year-old woman with diffuse cutaneous systemic sclerosis (SSc) complicated by precapillary pulmonary hypertension, interstitial lung disease treated with mycophenolate mofetil, and gastrointestinal involvement was admitted to our institution for hypoxemia and volume overload. Medical history included recent diagnosis of myopericarditis based upon cardiac magnetic resonance (MR) at an outside institution that was treated with tapering glucocorticoids (GC).

**Take-Home Messages**

• In patients with diffuse cutaneous SSc in acute heart failure, cardiac magnetic resonance should be considered.
• Endomyocardial biopsies often lack evidence of myopericarditis because the disease process is patchy not diffuse, and thus empiric treatment with glucocorticoids and CYC should be considered.

Workup at our hospital included chest computed tomography angiography that showed bilateral ground glass opacities, right upper lobe infiltrates, and no emboli. Transthoracic echocardiogram revealed global hypokinesis, left ventricular ejection fraction (LVEF) of 10%, pulmonary artery systolic pressure of 48 mm Hg and right atrial pressure of 10 mm Hg. The patient received intravenous (IV) diuretics, empiric vancomycin and piperacillin/tazobactam, and mycophenolate mofetil was held, given the concern for heart failure and pneumonia. Recurrent myopericarditis was suspected and IV methylprednisolone, 16 mg/d, was started. Antibiotics were discontinued subsequently, given the high probability that the chest infiltrates were edema rather than infection based on clinical course (eg, afebrile) and laboratory assessments (eg, negative respiratory pathogen panel and cultures). Cardiac MR demonstrated reduced biventricular function, 4-chamber dilation, LVEF of 11%, and focal late gadolinium enhancement in the subepicardial and midmyocardial muscle suggestive of myopericarditis (Figure 1A). Right heart catheterization with endomyocardial biopsy was pursued to evaluate for autoimmune myopericarditis given her prior history and unrevealing infectious workup: right atrial pressure of 10 mm Hg, mean pulmonary artery pressure of 39 mm Hg, and pulmonary capillary wedge pressure of 16 mm Hg, suggestive of mixed precapillary and postcapillary pulmonary hypertension. Five right ventricular endomyocardial biopsies demonstrated mild myocyte hypertrophy and hyaline arteriolosclerosis, but few inflammatory cells and no contraction band necrosis or fibrosis, results inconsistent with myocarditis (Figures 1B to 1D). Despite this, IV cyclophosphamide (CYC) 750 mg/m^2^ was initiated for a presumptive diagnosis of SSc myopericarditis. After clinical improvement, the patient was discharged on a tapering dose of prednisone 15 mg/d. Two weeks later, she was rehospitalized for heart failure with similar cardiac MR findings but improved with IV diuresis and was discharged on a tapering dose of prednisone 10 mg/d. After 2 months, the patient underwent a planned admission for IV methylprednisolone 1 g/d for 3 days followed by IV CYC 170 mg/m^2^ for myocarditis treatment. One month later, she received IV methylprednisolone 500 mg/d for 3 days followed by IV CYC 500 mg/m^2^. Cardiac MR then demonstrated a moderately dilated left ventricle, LVEF of 43%, normal myocardial stress perfusion, and improved enhancement. Transthoracic echocardiograms over the next 5 years revealed sustained improvement in biventricular function with LVEF of 30% to 40%. The patient had no further hospitalizations for heart failure during this time.

**Figure 1:**
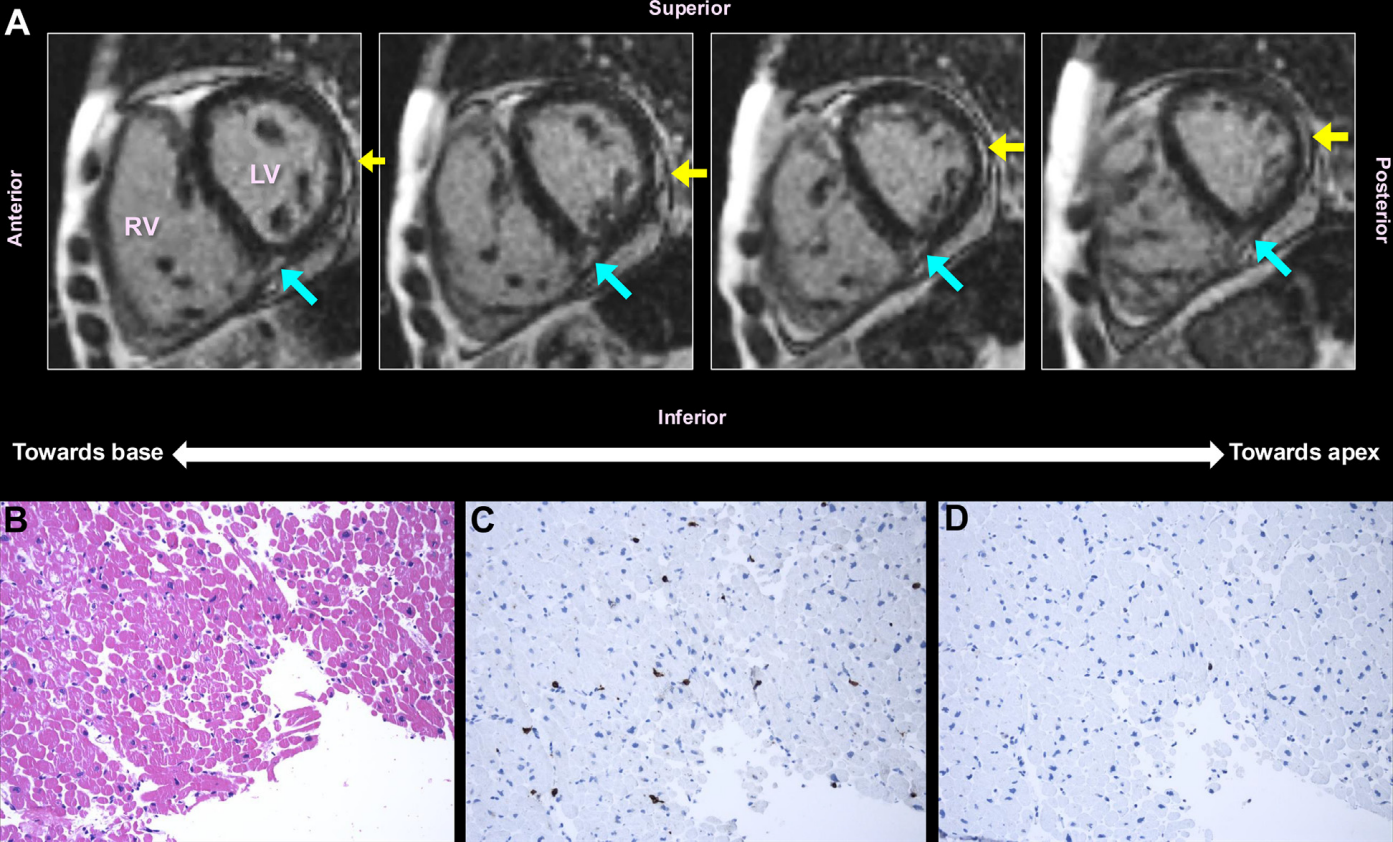
Presumed SSc Myopericarditis (A) Cardiac magnetic resonance showing short axis views of the patient's cardiac magnetic resonance with contrast. These images of short axis sections of the heart (basal to apical mid-myocardial segments) show evidence of prominent late gadolinium enhancement at the basal inferior septal insertion point (aqua arrows), which can occur in patients with RV strain. There is also prominent subepicardial delayed enhancement in the mid lateral to basal inferolateral walls (yellow arrows). Subepicardial LV myocardial enhancement is typically indicative of an inflammatory cause of cardiomyopathy (eg, myocarditis). (B to D) Cardiac histopathology (hematoxylin & eosin, CD3, and CD20, respectively) that fails to show inflammation with adjacent myocyte necrosis that is typical for myocarditis. LV = left ventricle; RV = right ventricle.

## Discussion

Primary cardiac disease as a direct consequence of SSc, or SSc cardiomyopathy, can manifest as microvascular coronary disease, systolic dysfunction, diastolic dysfunction, myocarditis, myocardial fibrosis, arrythmias, pericardial disease, and/or valvular disease.^1^ SSc cardiomyopathy has a reported prevalence of 7% to 39% and is associated with increased mortality.^2^ Despite this, little evidence is available to inform treatment of SSc cardiomyopathy. Herein, we present a patient with diffuse cutaneous SSc with acute heart failure. Extensive work-up supported a diagnosis of SSc myopericarditis. She received IV CYC and GC and achieved significant and prolonged recovery.

Our case is notable for our patient’s significant systolic dysfunction, the use of advanced imaging including cardiac MR for diagnosis, and our patient’s sustained response to therapy. SSc cardiomyopathy often presents insidiously, more commonly with diastolic versus systolic dysfunction as noted in our patient.^1^ Results of a EUSTAR (European Scleroderma Trials and Research) study noted a prevalence of SSc myocarditis-associated reduced EF of 5.4%.^3^ Although the transthoracic echocardiogram remains a mainstay for detection, cardiac MR, particularly with novel parametric techniques such as T1 mapping, is useful for characterizing myocardial inflammation, fibrosis, and/or perfusion abnormalities.^4^ Definitive diagnosis of inflammatory cardiomyopathy can be made with endomyocardial biopsy, typically demonstrating T lymphocyte infiltration and myocyte necrosis that are uncharacteristic of coronary ischemia. We suspect that our patient’s negative biopsy resulted from sampling error; Hauck et al^5^ found that >17 samples were needed to diagnose myocarditis in >80% of cases. Alternative inconclusive biopsy explanations include prior immune suppression or a noninflammatory etiology, yet cardiac MR findings and response to immunosuppression speak against this. Patients with myocardial fibrosis and progressive cardiac dysfunction may experience improved systolic function with moderate dose GC (<15 mg prednisone per day to be mindful of the potential for scleroderma renal crisis associated with high dose GC) and other agents.^6^ Previous myocarditis case reports have shown clinical and radiologic improvement with regimens including pulse dose GC, CYC, mycophenolate mofetil, or azathioprine.^7^^,^^8^ Despite her significant disease, IV GC and CYC resulted in lasting clinical and imaging improvement.

## Conclusions

We describe a young patient with severely reduced systolic function attributed to myopericarditis despite inconclusive endomyocardial biopsies. The case underscores the importance of multimodality imaging in diagnosis, characterization, and surveillance of SSc cardiomyopathy. A sustained response to immunosuppression treatment with prolonged follow-up is not reported elsewhere in the literature. This case adds to the growing body of evidence to guide treatment of SSc cardiomyopathy and myopericarditis.

## Funding Support and Author Disclosures

This work was supported by National Institute of Arthritis and Musculoskeletal and Skin Diseases grant [R01 AR073270] to Dr Hinchcliff; and National Heart, Lung and Blood Institute grant grants [U54 HL160273, NS R01 HL149423] to Dr Shah. Dr Hinchcliff has received consultancy fees from AbbVie and has received research grant support from Boehringer Ingelheim and Kadmon for investigator-initiated research projects; is a Scientific Advisory Board Member for the National Scleroderma Foundation; and has participated in clinical trials with Boehringer Ingelheim, AbbVie, Kadmon, Mitsubishi, Horizon, Emerald Health. Dr Shah has received research grants from AstraZeneca, Corvia, and Pfizer, and consulting fees from Abbott, Alleviant, AstraZeneca, Amgen, Aria CV, Axon Therapies, Bayer, Boehringer-Ingelheim, Boston Scientific, Bristol Myers Squibb, Cyclerion, Cytokinetics, Edwards Lifesciences, Eidos, Imara, Impulse Dynamics, Intellia, Ionis, Lilly, Merck, MyoKardia, Novartis, Novo Nordisk, Pfizer, Prothena, Regeneron, Rivus, Sardocor, Shifamed, Tenax, Tenaya, and Ultromics. All other authors have reported that they have no relationships relevant to the contents of this paper to disclose.
